# Supplementary material for: Micromixing Intensification within a Combination of T-Type Micromixer and Micropacked Bed
Source: Micromachines (Basel). 2022 Dec 24;14(1):45. doi: 10.3390/mi14010045 (PMC9866573; doi:10.3390/mi14010045)
Supplement: Supplementary file 1 [file micromachines-14-00045-s001.zip › micromachines-2033432-supplementary.pdf]

Electronic Supplementary Information

Micromixing intensification within a combination of T-type micromixer and micropacked bed

Zhou Lan, Yangcheng Lu*

State Key Laboratory of Chemical Engineering, Department of Chemical Engineering, Tsinghua University, Beijing 100084, China

*Corresponding author email: luyc@tsinghua.edu.cn

Data processing between I_3^- concentration and UV-Vis absorbance

The UV-Vis absorption spectrum of I_3^- was shown in Figure S1(a). The characteristic absorption peaks of I_3^- are 286 nm and 353 nm. And the peak at 286 nm may be affected by glycerol, so 353 nm was adopted as the best characteristic absorption peak of I_3^-. Deducting the influence of the corresponding background, we found a universal standard curve of I_3^- for the systems with different content of glycerol adapted in this work, and the fitting results were shown in Figure S1(b). The fitting equation was $y = 3.19 \times 10^{-5}x$. Therefore, combining the standard curve and equations (5), (8), and (9), the concentration of I_3^- can be detected by the online UV absorption spectrum, and the micromixing performance of the system can be quantitatively described by the segregation factor *X*s.

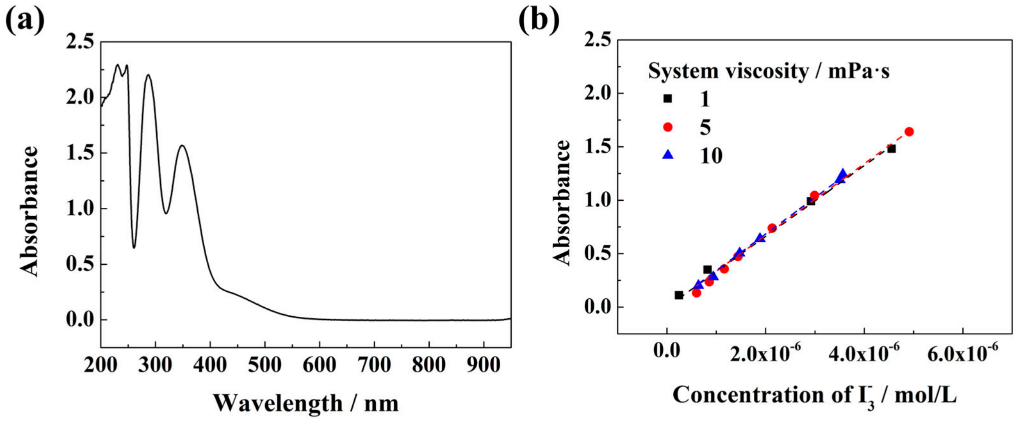

Figure S1. (a) UV-Vis absorption spectrum of I_3^-; (b) standard curve fitting results under different systems.

Data supplementation of micromixing performance within the microstructural combination

Figure S2 showed the influence of the length of micropacked bed on micromixing performance. When the length of the micropacked bed was extended from 5 cm to 7.5 cm, X_S of the system did not change much. This indicated that the mixing of the system has been completed after passing through the micropacked bed with a length of 5 cm.

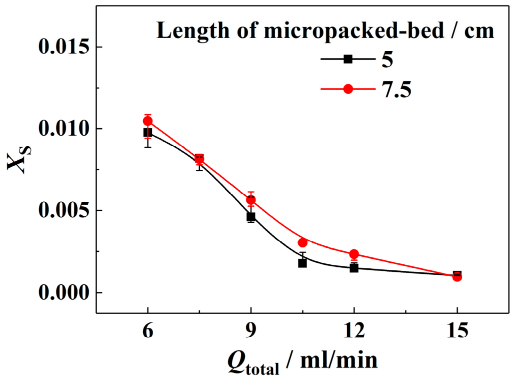

Figure S2. Influence of the length of the micropacked bed on the micromixing performance. Experiment conditions: micromixer, 0.25 mm of I.D.; connecting pipe, 0.5 mm of I.D., 5 cm of length; packing size, 0.177~0.250 mm; μ_A = 10 mPa·s, μ_B = 1 mPa·s; Q_A:Q_B = 5:1.

The influence of system viscosity on the micromixing performance when the ratio of flow rates (Q_A:Q_B) was 10:1 was illustrated in Figure S3(a). Compared with the ratio of flow rates of 5:1 (Figure 4(a)), it was found that the increase in the ratio of flow rates made the micromixing performance of the system worse, which was similar to the conclusion obtained in Figure 5. And when the viscosity of solution B was 1 mPa·s, the

influence of the viscosity of solution A was shown in Figure S3(b), which served as supplementary support for Figure 4(b).

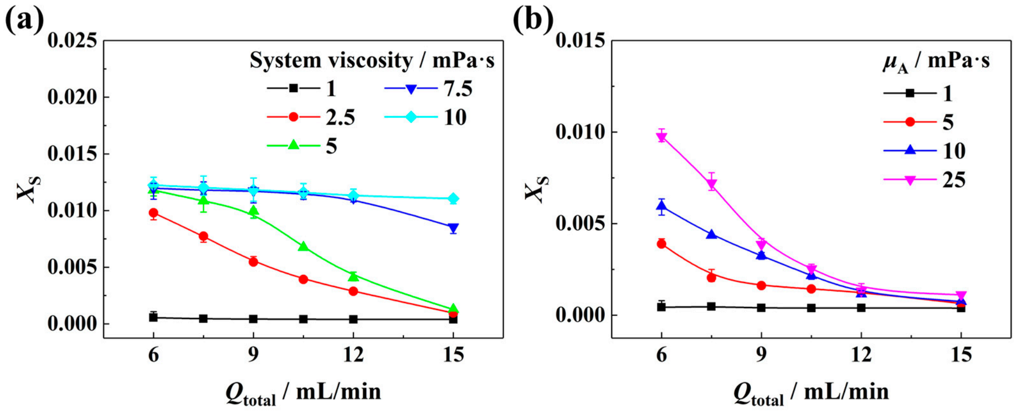

Figure S3. Influence of the fluids properties on the micromixing performance: (a) system viscosity, (b) the viscosity of solution A. Reference conditions: micromixer, 0.25 mm of I. D.; connecting pipe, 0.5 mm of I.D., 5 cm of length; micropacked bed, 5 cm of length; packing size, 0.177~0.250 mm. (a) $\mu_A = \mu_B$, Q_A:Q_B = 10:1; (b) μ_B = 1 mPa·s, Q_A:Q_B = 5:1.

Fitting results under different conditions

The fitting results of the correlation (Equation (10)) to some key influencing factors are shown in Figure S4, such as the I.D. of connecting pipe, system viscosity, and flow ratio. It can be seen that the correlation values agreed with the experimental data well within the ranges listed in Table 2. However, some individual points have large deviations due to the runout of the applicable range.

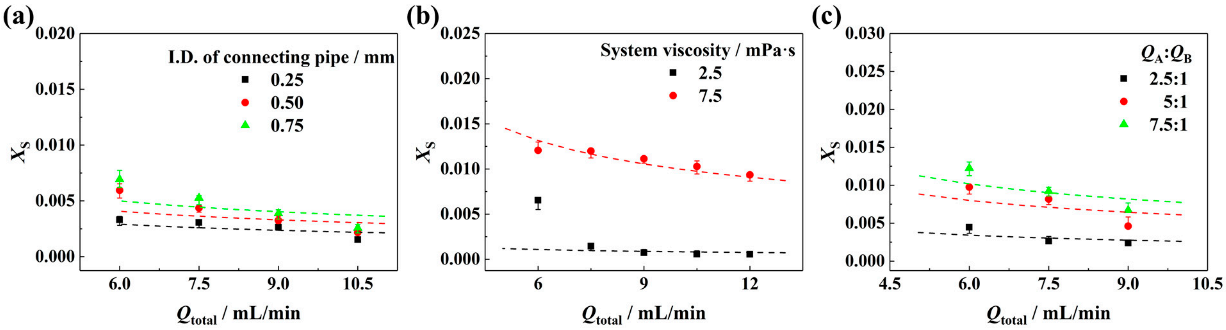

Figure S4. Experimental data (filled circles) and the correlation data (dashed line) under different conditions: (a) I.D. of connecting pipe, (b) system viscosity, and (c) flow ratio. Reference conditions: (a), (b) and (c) correspond to Figure 3(b), Figure 4(a), and Figure 5, respectively.
